# HastaLaVista, a web-based user interface for NMR-based untargeted metabolic profiling analysis in biomedical sciences: towards a new publication standard

**DOI:** 10.1186/s13321-019-0399-7

**Published:** 2019-12-05

**Authors:** Julien Wist

**Affiliations:** Chemistry Department, Universidad del Valle, Cali, 76001 Valle del Cauca Colombia

**Keywords:** Metabolomics, Metabonomics, Metabolic profiling, Untargeted analysis, Data analysis, Graphical interface, GUI

## Abstract

Metabolic profiling has been shown to be useful to improve our understanding of complex metabolic processes. Shared data are key to the analysis and validation of metabolic profiling and untargeted spectral analysis and may increase the pace of new discovery. Improving the existing portfolio of open software may increase the fraction of shared data by decreasing the amount of effort required to publish them in a manner that is useful to others. However, a weakness of open software, when compared to commercial ones, is the lack of user-friendly graphical interface that may discourage inexperienced researchers. Here, a web-browser-oriented solution is presented and demonstrated for metabolic profiling analysis that combines the power of R for back-end statistical analyses and of JavaScript for front-end visualisations and user interactivity. This unique combination of statistical programming and web-browser visualisation brings enhanced data interoperability and interactivity into the open source realm. It is exemplified by characterizing the extent to which bariatric surgery perturbs the metabolisms of rats, showing the value of the approach in iterative analysis by the end-user to establish a deeper understanding of the system perturbation. HastaLaVista is available at: (https://github.com/jwist/hastaLaVista, 10.5281/zenodo.3544800) under MIT license. The approach described in this manuscript can be extended to connect the interface to other scripting languages such as Python, and to create interfaces for other types of data analysis.

## Introduction

Metabolic profiling has been used to investigate physiological and pathological processes and to place them within the framework of both personalized and population healthcare. It has become a standard tool in the ‘omics’ family, delivering information on the products of gene-environment interactions and has been used to explore biomarkers and mechanistic aspects of both, chronic and acute diseases. Whilst applications of this technology to public and personalised health issues have met with some success, its full potential is limited by lack of standardisation of methodologies, which would enable sharing of resources, such as software libraries and datasets. Although thousands of documents have published human metabolic profiles, this mass of already existing terabytes of metabolic profiles are often not accessible to perform key tasks such as establishing a reference baseline of healthy patients. Publishing only figures and conclusions impedes the ability to re-use the data for further research. Reuse is necessary to review and reproduce studies and to accelerate innovation and discoveries as well as to prevent wastage of funding resources [1]. Some efforts worth mentioning are being made, for instance several databases allows users to share information about reference compounds or to upload their original data [2–4].

The hypothesis developed in this manuscript is that the kind of software involved in the data workflow influences the amount of effort that is required by one user to share his data in a way that is useful to others, with open-source pipelines presenting the lowest “activation barrier” and proprietary software the highest, often with an associated financial cost. On the other hand, the lack of user-friendly graphical interface available for current open-source software may discourage young researchers to explore their data or to move to commercial solutions. Improving current open source solutions to work with such data, therefore presents a high value: low energy proposition to overcoming the challenge of data analysis pipelines for acquisition and modelling of the current silos of metabolic profiles originating from disparate sources.

**Fig. 1:**
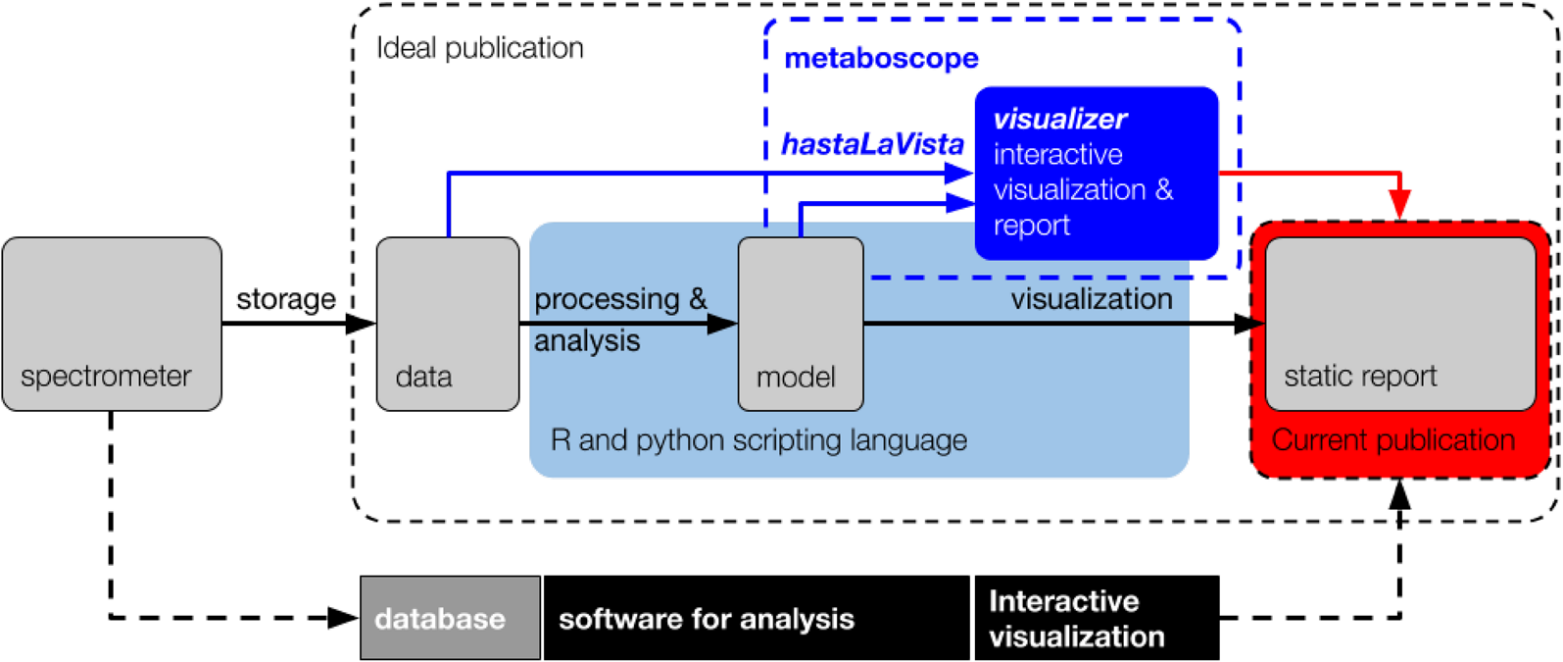
Schema of the data flow in metabolic profiling. Ideally, the traceability of the data at each step of the process is required and should be published. However, much proprietary software bypass this flow by hiding most of the internal processes and making it more difficult for the user to share their data adequately. The proposed solution (in blue) consists of a connector package, hastaLaVista, and several specific tools built on a javascript framework for visualization of data, *visualizer*. It provides a graphical user interface for R with similar features as found in proprietary software

Essentially, three components are required to manage the whole flow of data analysis: storage, computing (processing and analysis) and visualisation (see Fig. 1). Community-driven (open source) developments for storage of profiling nuclear magnetic resonance (NMR) or mass spectrometric (MS) data are available [2–6], some of which are aligned with the FAIR guidelines [7]. Open statistical analysis tools are also available but mostly oriented towards methodology-oriented researchers, offering maximum flexibility of analysis but requiring a certain level of programming skills. Examples include libraries in R [8] and Python [9]. This software is widely spread and supported by an active user community. Since both these platforms are used to test and showcase new methods and algorithms, they are usually ahead of commercially available software. Certain libraries also provide them with capabilities to visualize data, such as ggplot2 [10] and Matplotlib [11] to name only a few. In addition to the generic R and Python software packages, referred to above, some open developments offer comprehensive suites of dedicated metabolic profiling tools for the less-experienced users [12].

Open-source software manipulates data in open formats and ontologies [13] and provides scriptable capabilities that enable automated workflows that are well suited for sharing and reusing data. But most importantly, users have access to any part of the code if they need a deeper understanding of the data handling process, to modify its behaviour or to interoperate with other software.

On the other hand, proprietary solutions are designed for application-driven users in environments where there is an analytical need. Examples of such software include SIMCA (Umetrics, Umeå, Sweden), Prism (Graphpad, California, USA) and Chenomix (Chenomx Inc, Canada), which all are user-friendly and widely used, but require an initial outlay for the purchase of the software and, in some cases, an annual fee for licensing. This commercial software is by essence not intended to be repurposed by users or even modified. It is thus difficult to connect different proprietary tools into an integrated and automated platform, resulting in manual and fragmented workflows and multiple interim data versions that are difficult to share and reuse. Proprietary software is thus typically a “black box” that allows users to transform their spectra into reports, as illustrated in Fig. 1, but without exposing important internal processes for data handling. Usually, these software packages are dedicated to analysis and visualization (see Fig. 1), while some solutions offer storage capabilities like MestreNova (MESTRELAB RESEARCH, Santiago de Compostela, Spain), ACDLab (ACDlab, Toronto, Canada) and Chenomx. It is generally not apparent to the user if they provide a mechanism (API^1^) to import/export data from/to another system.

These proprietary solutions are praised by colleagues seeking simplicity and usability for their handy features and polished graphical interfaces. Indeed, visual inspection of the results of the different stages of the analysis is key to detect deviations from the idiosyncratic behaviour and avoid unfortunate conclusions. In general, visual inspection allows the user to detect and correct errors in the data workflow. However, in general, the more complex the programme, the less inclined the user is to explore and quality check model parameters.

Finally, in some cases, the data may become extremely large and complex demanding tremendous efforts to interpret and visualize them. Then, the traditional publication format (PDF, Portable Document Format) falls short in rendering this complexity to the readers and more complex and interactive solutions would be preferred for publication.

### The missing link

A weakness identified in all the common scripting languages that may discourage newcomers is the lack of an interactive graphical interface. Plots and annotations are mainly static.

One example of desired features would be the ability to hover or select a point in a projection (score plot) and display the corresponding spectra, or the other way round. Another would be the ability to pick a variable in a loading plot and see the result of univariate analysis. With R, this means modifying and running the script each time a new score or variable is selected, or displaying all the results at once. Although this is perfectly fine for skilled users, it remains tedious most users would prefer the polished graphical interfaces provided by commercial software. Indeed some packages aim to bring such interactivity in R such as Shiny (Rstudio Inc., Boston, USA), htmlwidgets [14], reactR (Facebook Inc., Menlo Park, USA), rChart [15]. User-friendly visualization is the missing link that impacts the use of open software and thereby the ability to share data for re-use.

### The link

The rapid growth of what has been coined “big data” increased the necessity for improved visualization tools. Interestingly, most of these are intended for the web as tremendous efforts were focused on making web browsers fast and reliable at rendering graphics. The *visualizer* package [16] developed within our research group also seeks to provide this link, but with an extreme rationale: all of the data workflows should be performed inside of a web browser, i.e., all the three components mentioned in the introduction (see Fig. 1) must be built in JavaScript. Although Javascript is a widely used and very well accepted framework to create interactive web applications, it has not yet earned its place as a scientific programming language. As a result, very few libraries are available for statistical analysis and the proposed solution only satisfies tech-savvy users. It is likely a mistake to believe that researchers will learn an additional language just because it provides a better graphical interface. A more suitable solution would use established tools to compute statistics and the *visualizer* framework to display the results interactively, i.e., to keep both computation and visualization separated.

Presented here is such a solution, exemplified using an NMR-based spectral dataset of characterising metabolic perturbations induced by bariatric surgery in a rat model for resolution of obesity and type 2 diabetes [17].

## Methods

### A generic framework

The first general concept behind the proposed approach requires to set a type for the information of interest and then create a module (function) that can display it seamlessly. This is not a new concept. In the object-oriented programming realm methods (functions) can be created that will act differently according to the kind (class) of the object that is input. Here we apply this same idea to visualization. For example an object can be defined of type “string” and value “C6H6” and will be displayed as “C6H6” while an object of same value but of type “molecular formula” will be displayed as “C_6_H_6_”. This is similar to changing the type of a column in a spreadsheet; if the chosen type is percentage, then typing “0.1” into a new cell will display “10%”. While the “string” and “number” type are common to most programming languages, more specific types, such as molecular formula are not.

This principle can be applied to more complex types of data. An object of type ”SMILES” [18] can be displayed as a chemical structure and objects of type “jcamp” may contain Infrared (IR) or NMR spectra to be displayed as chemist are used to: with reversed y or x-axis, respectively. Thus, for each new type of object a format must be chosen and a function created that can display the information as expected. Such a task is therefore greatly simplified if open format are available. Following this approach the objects can be created using any programming language, the most suitable one for data analysis, while the modules or function to display them may be built using another one, more suitable for that last purpose. It goes beyond the scope of this manuscript to list all the data types supported by *visualizer*, but most of them were implemented with the purpose of solving cheminformatics and bioinformatics problems: some demonstrations can be found elsewhere [19].

**Fig. 2:**
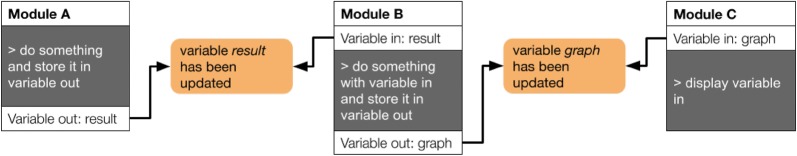
Each module is triggered by modifications of its input variable and can store the results of its operation into an output variable. This allows controlling complex data flows within a webpage

The second general concept is that each module function listens permanently to modifications of its input variables, unlike scripts that are run once. If an input variable is changed, the listening module executes itself and the information is displayed. In addition, the function may operate on the input data and store the result as a new variable, an output variable. For example, if *Module A* modifies the input variable of *Module B, Module B* will execute automatically upon notification (see Fig. 2). When it becomes necessary to perform more complex tasks, a module can apply a custom function to the input data and store the result in its output variable. The chaining of different modules allow the user to control complex flows of information and hence to build interactive graphical interfaces. Again, this is similar to defining a function in a cell of a spreadsheet, its result will be updated upon modification of the input cell.

A last important generic idea behind the *visualizer* is that data are often structured, i.e., a relation exists between different pieces of information. A molecule and a spectra may correspond to the same sample. This relation can be established by adding the same unique tag to all the objects that are related. Thus, a module can track the mouse position and herald the tag of the object that is being hovered. If another module has an object with the same unique tag it can highlight it.

The *visualizer* package implements the concepts described above, but despite the data analysis and visualization are kept separated, both are programmed in JavaScript.

### A connector tool

Acknowledging the fact that R scripting language is much more attractive for researchers in metabolomics than JavaScript, a connector is proposed here that ‘pushes’ the results (the objects) obtained with R into the *visualizer* specific environment for visualization, referred to as a *view* (*vista* in spanish).

**Fig. 3:**
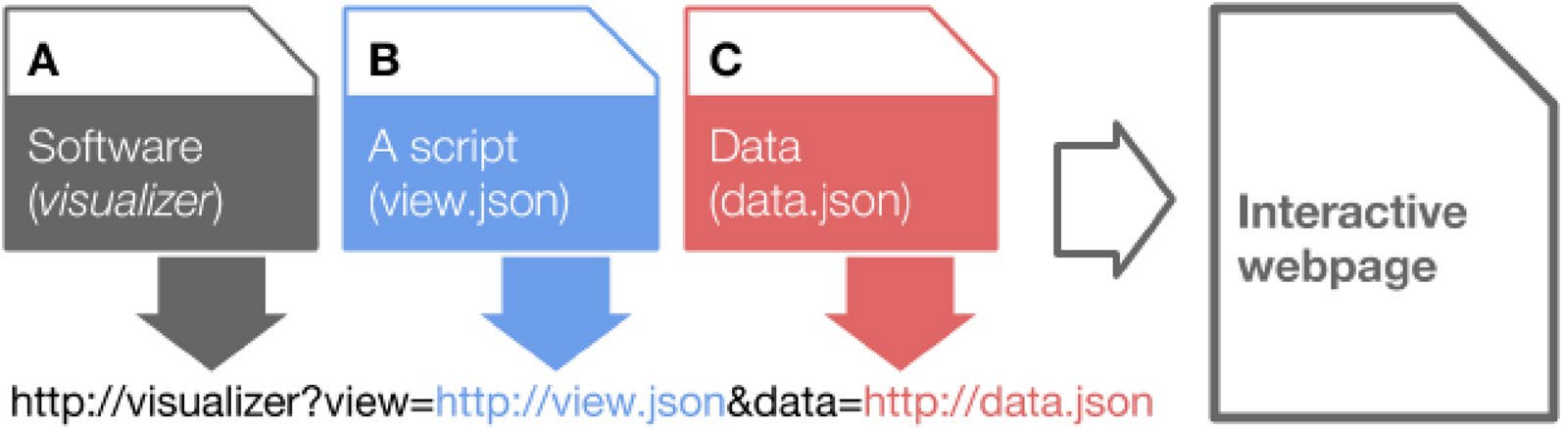
Schema of the *visualizer* system. Three elements are needed to display the information correctly, the app itself, the data and a file that contains the information about how to display the data. It is important to note that each element can be served by different servers within the restrictions imposed by security policies implemented in recent web browsers. Serving data locally reduces latency and ensures privacy while using globally served app and view files facilitate sharing

Thus, to visualize the results, 3 elements are required. The *visualizer* itself (Fig. 3A), a view.json file that contains the information about the visualization layout, i.e., the number, type, and position of the modules (Fig. 3B) and a data.json file that contains the result of the analysis, i.e., the data to be displayed (Fig. 3C). All three elements can come from different servers and their URLs concatenated as shown in Fig. 3.

A package for R, called *hastaLaVista* [20] does just this: it creates a data.json file, allows the user to select a view.json and make them both available to a local instance of the *visualizer* framework using the *servr* package [21]. The execution of an R script automatically launches the browser and points it to the chosen view and data URL as described in Fig. 3.

### A specific interface

View files are generic for scholars of the same research field, that is to say, most users in a given field display and interact with their data in a similar manner. For example, models obtained by multivariate analyses are interpreted by looking at a score plot or a similar projection plot and at a loading plot or its equivalent, representing the sample and variable space respectively. Thus, the same view.json can be used to interpret results obtained using PCA, PLS, or O-PLS-DA and the like. In contrast, the input data are different and are prepared differently for each analysis. It is not uncommon for the same dataset to be submitted to several processing treatments (baseline correction, referencing, alignment, binning, etc.) prior to input.

Thus, pre-established *view* files are specifically designed for the untargeted analysis workflow and are included within the *hastaLaVista* package, as an example.

## Results

**Fig. 4:**
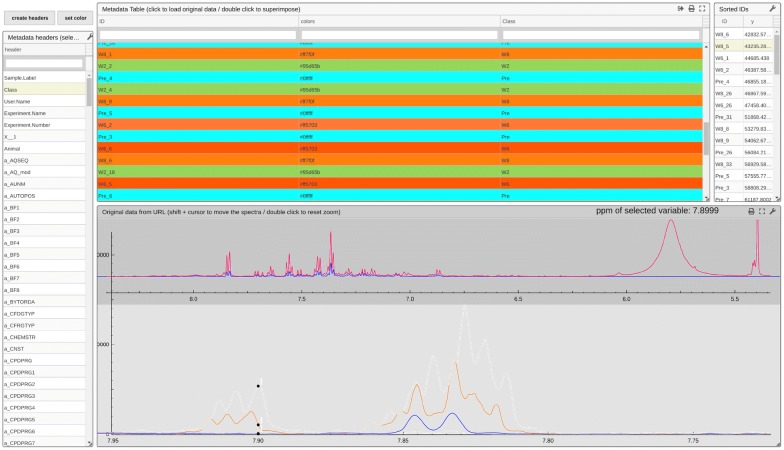
*dataExplorer* enables the user to explore the data and check for sources of experimental noise. This view allows the data to be colored according to any registered metadata. The *visualizer* also supports overlaying the original spectra to evaluate the effect of pre-processing, exemplified here for removal of the water signal and normalization by PQN to decrease concentration difference effects. The white traces in the bottom window represent the skyline and bottom-line (maximum and minimum intensity values respectively for each data point). Selecting a point (here at 7.89 ppm) will sort the samples in the table on the right according to the intensity for that variable, expediting the identification of samples with major concentration

The *hastaLaVista* package and several specific tools or *views* are presented here that may assist with untargeted multivariate analysis, as an illustration of the proposed methodology. The first one, *dataExplorer,* allows visual inspection of the data. Figure 4 shows its application to a dataset of 59 spectra acquired by Li et al. [17] from urine samples obtained from rats (see Additional file 1).

Provided with the following information: a unique identifier index, the actual data (spectra), optional metadata and the URL of the original spectra in JCAMP format, *dataExplorer* allows spectra to be coloured according to metadata (see Fig. 4). The table on the left allows the selection of the metadata that should be used for coloring and which are displayed in the main table (top-middle). This table enables users to select data, which results in visualisation of the corresponding spectrum. The top lines relate the actual data used in R (blue line) with the original file in JCAMP format (red line). This is useful to check the pre-processing of the data, e.g., to identify experimental problems (poor shims, contamination during sample recollection, etc.), to check the calibration and facilitate alignment of the data.

It can be observed that the water signal has been trimmed (it is not present in the blue line) and that the data have been normalized to take inter-sample differences in concentration into account, using a standard Probabilistic Quotient Normalization (PQN) approach [22]. The bottom traces present the selected spectra colored according to the chosen metadata, here according to the classes that are later used for the multivariate analysis (relating to a surgical intervention). Skyline and bottom line (maximum and minimum intensity values respectively for each data column) are shown in white. Selection of a variable sorts the spectra in order of ascending intensity in the table on the right. This enables the quick identification of traces with characteristic features. Here, samples 5 and 6 at week 8 are found with unexpected signals between 7.8 and 7.95 ppm, previously observed in rats after ingestion of bedding material.

Although the results are displayed as a web page, this latter is served locally, i.e., the system works offline and requires no internet connection. It also means that no data are uploaded elsewhere, thus keeping the information private. Moreover, these data are often voluminous and upload/download steps would add unnecessary delays to the analysis. Since the data matrix is served from R to the *visualizer* webpage, there is an upper limit to the size of the data imposed by the browser. This limit can be overcome by substituting each spectrum by its URL. This ensures that only spectra selected for display are indeed loaded into memory. They can be pulled from a laboratory database to avoid local copies of sensitive datasets.

**Fig. 5:**
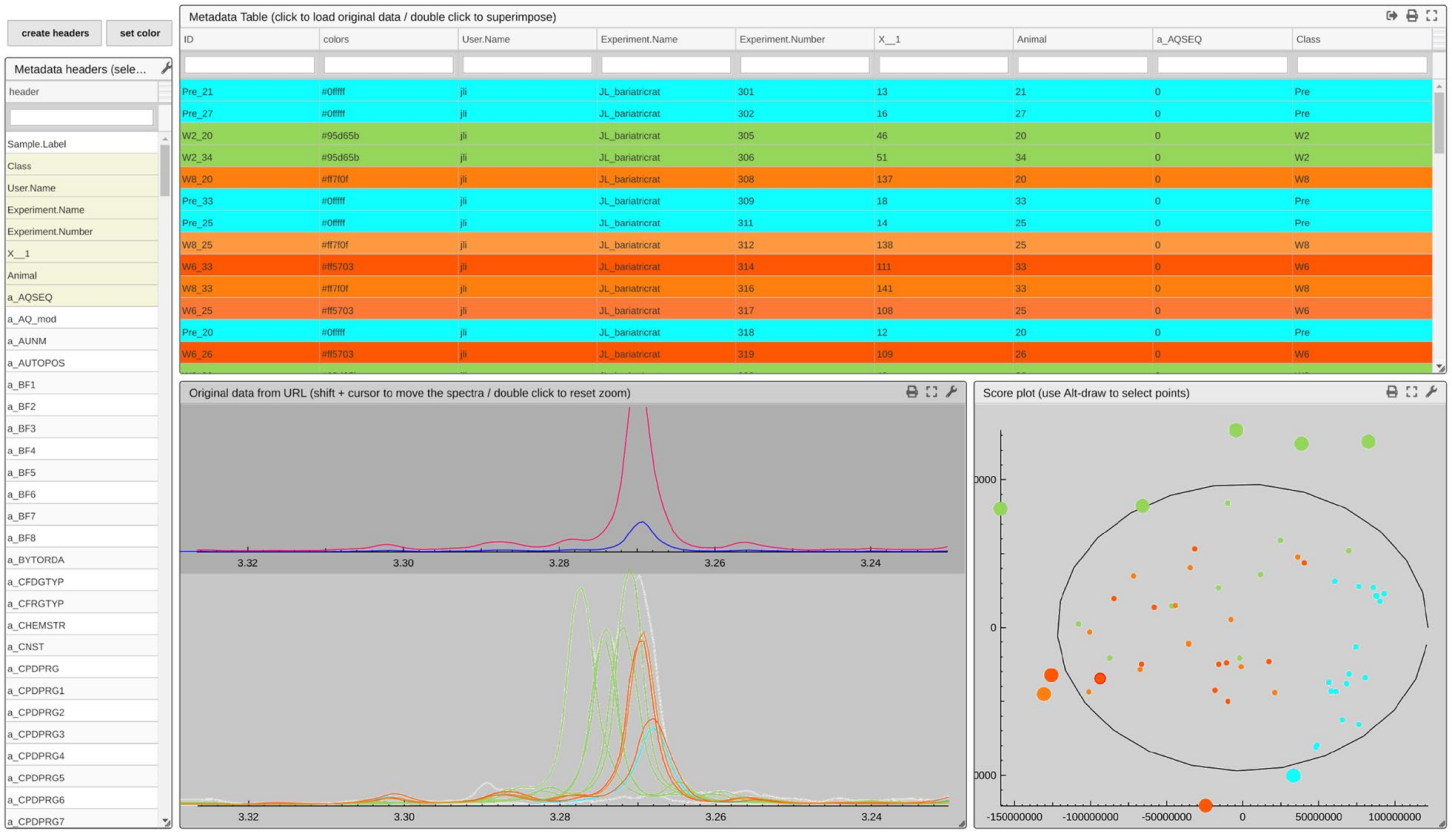
The *dataExplorer 1.1* view. The main goal, here, is to understand why a sample deviates by looking back at the data. Superimposing regular data on top makes easier to spot differences. A lasso tool permits selecting scores and displaying their spectra. Outliers selected at the top of the score plot (large green circles) exhibits the highest concentrations in Trimethylamine-N-oxide in rats 8 weeks following bariatric surgery

The second step involves performing a simple multivariate analysis such as PCA (principal component analysis) to identify samples that deviate, termed ‘outliers’. A researcher expects the ability to select outliers from a score plot and to return to their corresponding spectra where the idiosyncratic spectrum can be superimposed on ‘well-behaved’ data for comparison. The R script must compute the scores and provide the associated spectra and metadata. The *dataExplorer 1.1* view (see Fig. 5) will display the scores colour-coded according to the chosen metadata. A lasso tool permits the selection of points in the ‘scatter plot’ and displays their corresponding spectra. Hovering over the table allows one or several spectra to be displayed. Since there is a link between each score and its row in the table, clicking a row in the table will highlight the corresponding score in the scatter plot. This view is particularly useful at an early stage of the modelling analysis to track sources of experimental noise, such as batch effects, or errors induced by different operators (see Additional file 1). Here, it can be observed that green ‘outliers’ representing a subset of the urine samples obtained 8 weeks post-operation are exhibiting the highest urinary concentrations of Trimethylamine-*N*-oxide (TMAO) and also show a systematic positional shift in indicating an increase in urinary pH. Raised pH has been reported for a subset of bariatric patients who developed renal stones post-surgery [23]. Although previous papers using metabolic profiling to characterize the metabolic consequences of bariatric surgery have reported altered TMAO excretion, the view allowed easy interaction to identify increased excretion, coupled with the altered shift for the subset of week 8 animals who appeared as outliers in the plot.

**Fig. 6:**
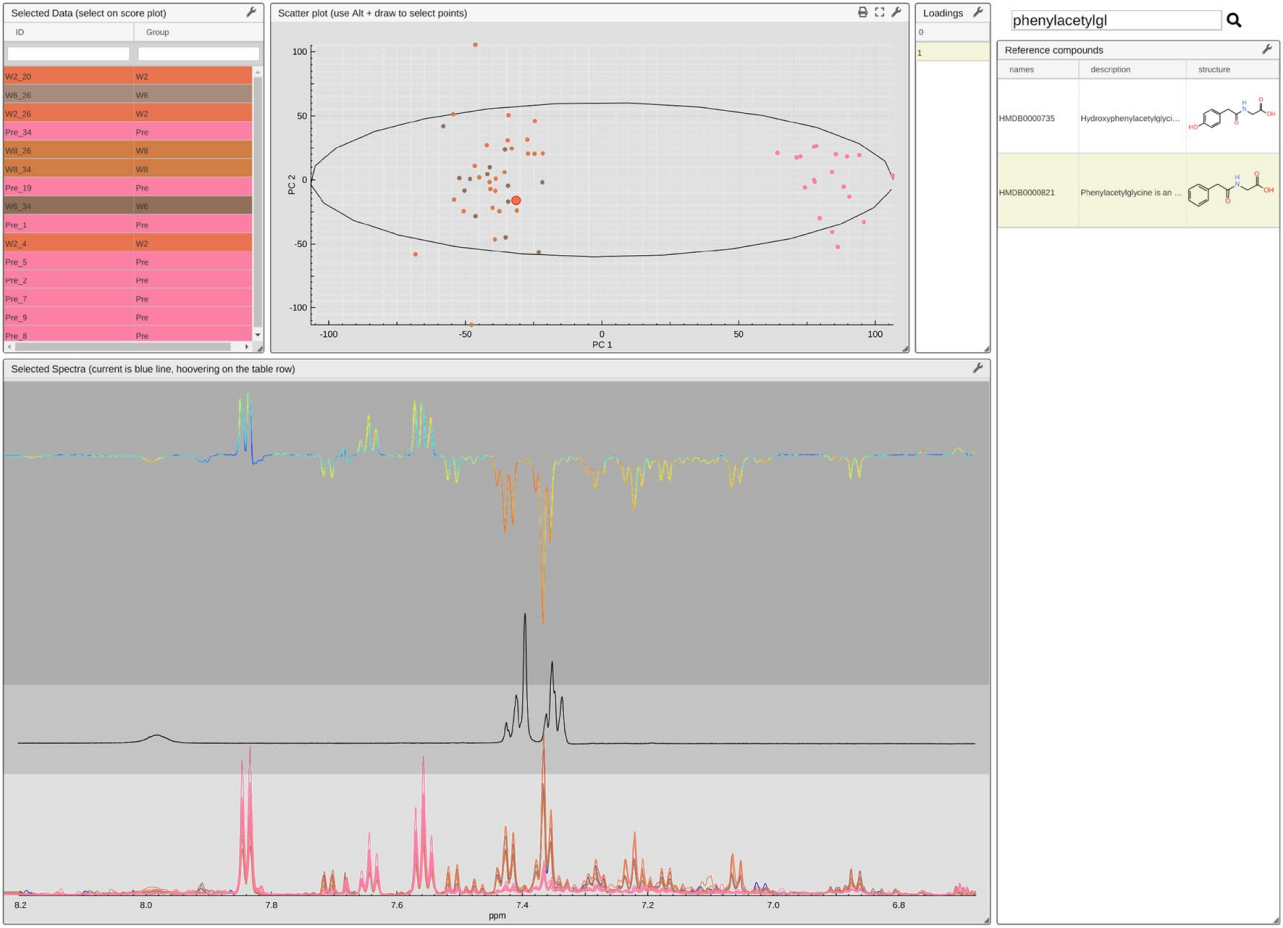
The *metaboscope* view allows a user to display scores and loadings. Reference compounds can be searched and their spectra superimposed to help with identifying putative metabolites. Here, scores were obtained using OPLS. The loading trace is back calculated, as the covariance of the score with the data and are coloured-coded according to the correlation. Here, the reference spectrum of PAG (phenylacetylglycine) is displayed

The last crucial step in untargeted analysis is the identification of putative metabolites [24]. Here, the *metaboscope* view (Fig. 6) expects scores, loadings and a colour-code for loadings, in addition to the unique identifier, group and associated data (see Additional file 2). Three *spectra displayer* modules are overlaid to display the data (bottom), the spectrum of a reference compound (middle) and the colour-coded loadings (top). The x-axis zoom function of the three modules is synchronized to ensure that all three can be aligned. An internet connection is required to retrieve and display a list of reference compounds aggregated from different open repositories such a BMRB [2] and HMDB [3]. Compounds may be searched by name or id. Once selected, the reference spectrum will be retrieved from our online database. In certain cases, HMDB does not provide original data but only a list of peak. Then, the database automatically reconstructs an artificial spectrum with perfect and noiseless baseline. Here, the reference spectrum of PAG (phenylacetylglycine) confirms the importance and presence of this metabolite in the samples that showed an inverse correlation with cell survival in in vitro studies [17].

**Fig. 7:**
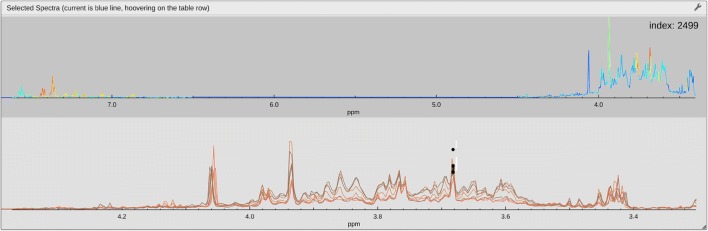
The lower portion of the *scoresExplorer 2.0* view showing two overlaid spectra displayer modules. The top module depicts the selected spectra colour-coded using the row of the correlation matrix corresponding to the selected variable (black dots)

An additional tool, *scoresExplorer*, enables interacting with STOCSY (Statistical TOtal Correlation SpectroscopY) data [25]. Selecting a point in the spectra (see Fig. 7, black dots), here the variable with index 2499, triggers the display of the selected spectrum colour-coded with the row 2499 of the correlation matrix, or any other statistics computed in R (see Additional file 3). Here, the selected variable corresponds to a singlet that is expected to belong to PAG, which is confirmed by the high correlation (red coloration) with the multiplets in the aromatic region of the spectra.

**Fig. 8:**
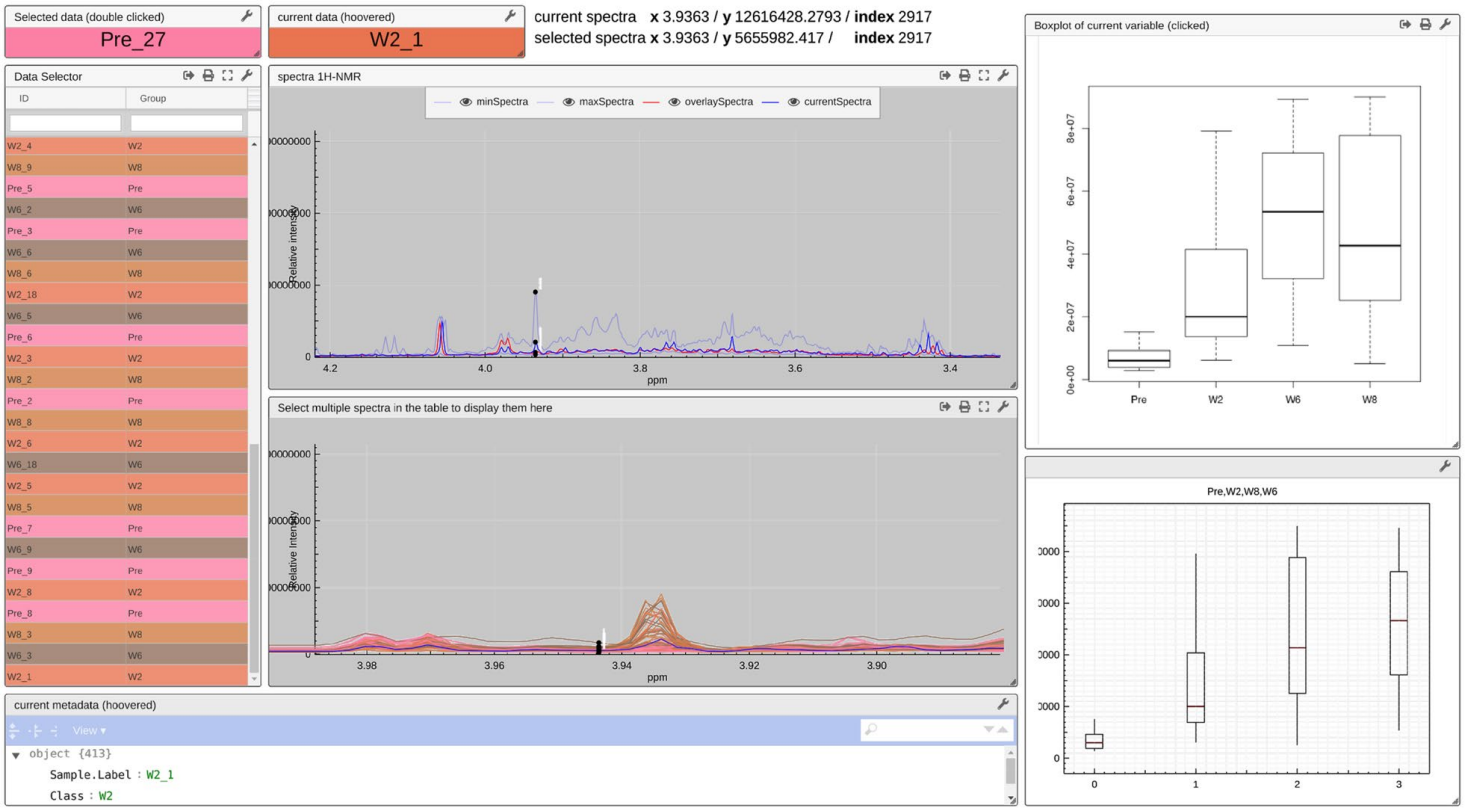
The *univariateExplorer* view allows to browse and inspect the data to evaluate the applied preprocessing strategy. Here we can check the spectra and observe that creatine has an interesting behaviour according to univariate analysis

Once the multivariate analysis is performed and important variables and compounds are identified, the *univariate* view (see Additional file 4) displays univariate statistics interactively, as illustrated in Fig. 8. The tool is provided with a unique identifier and group information and, in this case, with boxplots for each variable computed in R. Hovering over the table cell will display the corresponding spectrum (blue curve) and the metadata. Clicking on a row of the table allows superimposing a second spectrum (red curve). Hovering over the spectra allows selecting a variable. Clicking on this latter will retrieve and display the pre-calculated image (in png format). In addition, for the sake of showcasing the flexibility of the proposed solution, the view re-calculates univariate statistics for the selected samples, displayed in the module in the right bottom corner. Here, the selected variable corresponds to creatine that is related to muscle turnover.

## Discussion

This example illustrates well the balance that has to be reached between interactivity and ease of programming. Either the computation is done on the fly and allows to recalculate when samples are selected or unselected, but this has to be programmed using JavaScript, or the computation is performed in R using any new fancy package available, but offering less flexibility, e.g. to remove outliers without having to run the R script again.

Since all the code required for the views to operate is contained within the view, these can be readily modified by researchers. Each module can be moved and resized like a regular window and its behaviour adjusted using the parameter icon on the top right corner or using the right-click menu. To make the modifications permanent, the content of the view.json file can be copied from the web page and saved as a new file.

This manuscript demonstrates the potential of JavaScript and dynamic web pages as graphical interfaces for scientific data analysis. Similar to using code written in C to accelerate computation in higher level programming languages such as R and Python, JavaScript libraries may be included to improve graphical rendering capabilities. Although this example is built around R scripting language, all the *hastaLaVista* R package does is to format a data.json file. It would be trivial for Python *aficionados* (or of other programming languages) to produce such a data structure (data.json) and use the very same graphical interface (view.json).

The solution described in this manuscript is a proof-of-concept of a new kind of publication that, in the future, may complement or even replace the traditional PDF format. The advantage of this approach is that data are being published instead of shared, this is a subtle but important difference. Shared data are considered a complement to publications, while they are truly the core of it.

## Conclusions

We demonstrate a new approach that makes possible the interactive analysis of metabolic profiling data applying it to the mechanistic investigation of Roux-en-Y gastric bypass (RYGB) surgery in a rat model [26]. The described software complements the standard R workflow analysis by providing a dynamic graphical interface to visually explore the data and identify: (i) compositional differences before and after an intervention (e.g. surgery); (ii) identify outliers and individuals who behave idiosyncratically; (iii) visually inspect multivariate models and validate the effects of the intervention. Thus, the proposed software provides a user-friendly and interactive interface that can be adopted regardless of the experience level of the user and that can be adapted to work with other analysis pipelines. More experienced users can easily modify and adapt the current tools for their specific needs, for instance, this same concept can be applied to mass spectrometry data. Therefore, this approach represents a proof-of-concept for a new kind of publication, where the data and the analysis would be part of a single document.

## Supplementary information

**Additional file 1.** hastaLaVista data explorer. R script that illustrates how to use hastaLaVista to explore data according to metadata.

**Additional file 2.** hastaLaVista *metaboscope* view. R script that illustrates how to use hastaLaVista to explore results from multivariate analysis including retrieving spectra from reference compounds.

**Additional file 3.** hastaLaVista scores explorer. R script that illustrates how to use hastaLaVista to explore univariate scores computed by multivariate analysis.

**Additional file 4.** hastaLaVista univariate explorer. R script that illustrates how to use hastaLaVista to explore univariate statistics computed for each variable.

## Data Availability

Example R scripts are included in this published article and its additional files. The R package can be found as described below. Project name: hastaLaVista Project home page: https://github.com/jwist/hastaLaVista Operating system(s): Platform independent Programming language: R and JavaScript License: MIT.

## Footnotes

1 Application programming interface. A set of standardized functions that a user can expect for a task.
